# Trauma-focused cognitive behavioural therapy for young children: clinical considerations

**DOI:** 10.1080/20008198.2018.1433929

**Published:** 2018-03-15

**Authors:** Elisabeth Pollio, Esther Deblinger

**Affiliations:** CARES Institute, Rowan University School of Osteopathic Medicine (RowanSOM), Stratford, NJ, USA

**Keywords:** Childhood trauma, TF-CBT, young children, preschoolers, caregivers, developmental considerations, trauma en la infancia, TCC-CT, Niños pequeños, Preescolares, Cuidadores, Consideraciones sobre el desarrollo, 童年创伤, TF-CBT, 幼儿, 学前儿童, 照顾者, 发展性考虑, Young children and their caregivers are highly responsive to TF-CBT. To implement TF-CBT with this population most effectively, it is important to consider children’s developmental level and implement the model accordingly. The clinical strategies described above are suggested applications to enhance young children’s outcomes in the aftermath of trauma.

## Abstract

Trauma-focused Cognitive Behavioural Therapy (TF-CBT) has been utilized with children of a wide age range and with diverse trauma experiences. This article will focus on the application of TF-CBT to young children. After presenting an overview of the model, challenges and developmentally-sensitive and creative strategies for engaging young children and their caregivers in TF-CBT PRACTICE components will be highlighted. A brief review of the strong empirical support for TF-CBT will then be provided.

## Abstract (es)

La Terapia Cognitiva Conductual centrada en el trauma (TCC-CT) se ha utilizado con niños de un amplio rango de edad y con diversas experiencias traumáticas. Este artículo se centrará en la aplicación de TCC-CT a niños pequeños. Después de presentar una descripción general del modelo, se resaltarán los desafíos y las estrategias creativas y sensibles al desarrollo para involucrar a los niños pequeños y sus cuidadores en los componentes de PRÁCTICA de TCC-CT. A continuación, se proporcionará un breve análisis del fuerte apoyo empírico para la TCC-CT.

## Abstract (zh)

创伤中心的认知行为疗法（TF-CBT）使用于广泛年龄段和多种创伤经历的儿童。本文将集中讨论在幼儿中应用 TF-CBT。在综述这个模型之后，将重点指出让幼儿及其照顾者参与到 TF-CBT 实践中遭遇的挑战、发展有关的策略和创造性方法。接下会提及到支持TF-CBT的强有力的实证证据的综述。

Trauma-focused Cognitive Behavioural Therapy (TF-CBT; Cohen, Mannarino, & Deblinger, 2017; Deblinger, Mannarino, Cohen, Runyon, & Heflin, 2015) is an evidence-based treatment for childhood trauma that has been applied to children with diverse trauma experiences across a wide age range. TF-CBT is a components-based treatment model for children who have experienced trauma and their nonoffending caregivers. The model involves spending session time with children individually, caregivers individually, and with children and caregivers together (conjoint sessions). TF-CBT engages children and their caregivers in a gradual exposure process that begins at the start of treatment with acknowledgement of the traumas experienced, the learning of skills to cope with trauma reminders and other stressors, and ultimately making meaning of the traumas experienced. TF-CBT consists of three phases: stabilization and skill building, trauma narration and processing, and integration and consolidation of lessons learned. The components of TF-CBT can be summarized by the acronym PRACTICE: **P**arenting and psychoeducation, **R**elaxation, **A**ffect expression and modulation, **C**ognitive coping, **T**rauma narration and processing, **I**n vivo mastery, **C**onjoint sessions, and **E**nhancing safety and future development. Children and caregivers typically move through the components in parallel with caregivers serving as role models and supports for skills being acquired. Depending on the complexity of the presenting concerns, treatment is typically completed in 8–20 sessions.

## 1. TF-CBT as applied to young children

TF-CBT may be utilized when there is evidence of trauma and a possible link between the trauma and the child’s symptoms. TF-CBT is appropriate for children ages 3–18 years who have at least some memory of the trauma and their nonoffending caregivers. Single or multiple-incident trauma(s) as well as complex trauma may be addressed using TF-CBT. Exclusionary criteria include active suicidality, dangerous acting out behaviours, very brief placement (although children in foster care, unless in an emergency or other very brief placement, are appropriate), and active substance abuse on the part of the child or caregiver. Of note, once unsafe behaviours stabilize or substance abuse is treated, TF-CBT may be appropriate.

In addition to these more general criteria, it is important to assess young children’s verbal capacity and memory of the trauma prior to initiating TF-CBT. This may be done by eliciting an account of a positive/neutral recent experience and then eliciting a baseline trauma narrative as described in Deblinger and colleagues (2015). To participate successfully in TF-CBT, young children should be able to provide at least a brief acknowledgement of the trauma(s) to be addressed and demonstrate an ability to share a narrative about a neutral experience with some details.

Due to their limited attention span, total session time with young children may be 20–30 minutes with the remainder of the session devoted to work with nonoffending caregivers. It is recommended that young children be engaged for short bursts of activity (5–15 minutes) of focused skill building or trauma narration with planned positive activities that give children a break while incorporating fun in the therapy exercises. The repetition of skills is critical for acquisition, particularly with young children.

Though nonoffending caregivers are strongly encouraged to participate in TF-CBT regardless of the child’s age, primary caregivers are young children’s most influential role models and thus are critical to the TF-CBT process. As young children learn a great deal through observing and interacting with caregivers, it is essential for caregivers to develop effective coping and parenting skills through participating in TF-CBT. Research has documented that caregivers’ levels of abuse-related distress strongly predicted young children’s emotional and behavioural responses to treatment. Parental support became an even greater predictor of children’s post-abuse adjustment during the year following treatment (Cohen & Mannarino, 1998). These findings highlight the important influence of nonoffending caregivers on young children’s response to treatment.

### 1.1. Parenting

Research has revealed important strategies for engaging nonoffending caregivers in TF-CBT (Dorsey et al., 2014). Primary caregivers of young children determine whether children receive therapy and whether they successfully complete or drop out of treatment. It is therefore essential that caregivers understand the rationale for treatment and specific ways in which their participation will help their children develop/regain their ability to regulate emotions and process the traumas endured. To ensure consistent attendance, it is important to identify and resolve concrete obstacles to participation. For example, it is important to problem-solve how young children will be cared for while caregivers participate in treatment. It is ideal to provide oversight in the waiting room for young clients and their siblings; when onsite childcare is unavailable, other arrangements can be made such as scheduling caregiver sessions when children are in school.

Research suggests that more important than concrete obstacles are attitudinal obstacles to treatment (McKay & Bannon, 2004). Thus, it is helpful to inquire about prior therapy experiences with caregivers and explain the benefits of TF-CBT. If caregivers indicate prior treatment did not adequately address behavioural concerns, it is important to clearly describe how skills learned in TF-CBT will reduce the likelihood of ongoing behaviour problems and explain how TF-CBT sessions will help caregivers create/re-establish a structured and nurturing home environment that can assist young children in coping more effectively.

It is critical to work with caregivers on positive parenting skills, as the effective use of praise, positive attention, and other rewarding consequences for adaptive behaviours are more effective long-term than relying on negative consequences for problematic behaviours. Negative consequences alone do not work in the absence of a nurturing/predictable environment. When negative consequences are necessary, it is important those consequences are mild and brief for young children (e.g. 5-minutes in time-out). For both positive and negative consequences, the first few minutes following a behaviour has the most impact on a young child’s future behaviour, as it helps the child directly connect the consequences with the behaviour.

### 1.2. Psychoeducation

Young children’s emotions and behaviours in the aftermath of trauma can be worrisome to their caregivers. However, caregivers often have little understanding of child development leading them to overreact to normative transient fears and noncompliant behaviours. Thus, a critical aspect of psychoeducation with caregivers of young children is a review of their children’s emotions and behaviours with a focus on differentiating between problematic and normative behaviours. Some fears (e.g. monsters), sexual behaviours (e.g. touching one’s private parts), and noncompliant behaviours are common in young children. Noncompliance that appears or increases following trauma may reflect young children’s natural development and growing interest in control rather than trauma reactions. When caregivers do not overreact to normative fears/behaviours, they dissipate as children outgrow them. In the aftermath of trauma, however, caregivers may be particularly vulnerable to interpreting new behaviours as trauma reactions. For example, child sexual behaviour after an experience of sexual abuse may be misinterpreted as a reflection of the trauma. Undue attention to normative sexual behaviours can cause them to increase in intensity, frequency, and duration, thereby increasing the potential for them to become problematic. Thus, it is important to educate caregivers about common reactions to trauma as well as normative behavioural, emotional, and sexual development (Friedrich, Grambsch, Broughton, Kuiper, & Beilke, 1991) and help them respond effectively (Allen & Armstrong Hoskowitz, 2017; Deblinger et al., 2015).

Psychoeducation for young children is essential and should be provided in a developmentally-appropriate manner. Therapists may take note of the number of words children use in their verbal communications. Though challenging, it is helpful for clinicians to communicate in similarly simple 4–6-word sentences. Clinicians should also be aware of young children’s tendency to imitate words/expressions without fully understanding their meaning. A common error made with young children is use of the word ‘fault.’ While young children may imitate adults who say the trauma/abuse was not their fault, they may have little understanding of the meaning. Thus, it is important to address the issue of responsibility for abuse in more developmentally-understandable ways. Asking children, for example, ‘Who made the abuse happen?’ may be a better way of initiating a conversation that may alleviate children’s feelings of responsibility. Helping young children make a list of reasons children are not responsible for adult behaviours may make the concept of responsibility more concrete.

Given young children’s tendency toward concrete thinking, it is useful to share images when conveying information regarding the prevalence of the experienced trauma(s). Rather than offering a percent, it may be more helpful to show pictures drawn by other children who experienced the same trauma(s) or show about how many children in a picture of children are likely to have experienced the same trauma. In general, it is important to not overload young children with too much information. Picture books for young children can convey important information about the specific trauma(s) at the appropriate developmental level. To assess whether children are retaining information provided, it can be helpful to ask open-ended questions, while using reflective listening to reinforce what children are expressing. Even preschool children are capable of correcting therapist’s reflective listening responses if they think they were misunderstood.

### 1.3. Relaxation

Young children and their caregivers can benefit from learning relaxation strategies. Focused breathing can be learned by young children; for example a music video was developed by Sesame Street to teach preschoolers how to manage negative feelings with belly breathing (https://nj.pbslearningmedia.org/resource/sesame-belly-breathe/belly-breathe-sesame-street/#.WZjsLCiGN9M). Young children are also capable of successfully using their imagination to manage tension. Therapists can help children visualize relaxing scenes by drawing pictures and/or describing images of comforting scenes to use when feeling anxious. Young children also respond well to images (e.g. the beach) and/or cue words (i.e. breathe) to help them physically relax their bodies. Images of uncooked and cooked spaghetti, for example, can help children learn to tense and relax. Having control over bodily sensations is reassuring to children whose traumatic experiences may have left them feeling unable to control much of anything including, in the case of sexual abuse, access to their bodies. Caregivers are encouraged to support children’s effective age-appropriate efforts to manage tension in their bodies (e.g. hugging a teddy bear), while discouraging the use of age-inappropriate and/or ineffective strategies (e.g. punching a pillow). Young children are also skilled at engaging in mindfulness as they naturally engage fully in the present moment. There are wonderful books designed to reinforce children’s mindfulness skills (e.g. *Peaceful Piggy Yoga*; MacLean, 2008).

### 1.4. Affect expression and modulation

Young children’s emotional vocabulary may be limited but they have the capability to expand it. Even preschool children are typically able to identify the primary emotions of happy, sad, mad, and scared. Young school-age children often start to understand other emotions including worried and surprised as well as the concept that emotions have different degrees. When teaching skills to young children, concrete examples and engaging activities are most helpful. For affect expression, children can review pictures of people with different facial expressions or draw feelings faces. The therapist and child can play ‘feelings charades’ in which one person demonstrates a feeling through facial expressions and body language (without speaking) that the other has to guess. To explain the different degrees of feelings, a feelings thermometer can be used. As children expand their emotional vocabulary, they are encouraged to express their feelings through words rather than behaviours. This is particularly important as children who express feelings in words have been shown to exhibit more positive adjustment (Eisenberg, Cumberland, & Spinrad, 1998). Caregivers may be asked to praise children’s efforts to express their feelings in words between sessions. While the focus during this component is generally not trauma-related, for gradual exposure purposes the therapist may ask the child to circle feeling faces or words experienced during the trauma(s).

Positive activities have been associated with improvements in mood (Layous, Chancellor, & Lyubomirsky, 2014). The therapist and child can generate a list of activities the child enjoys and include pictures of each activity so the child can easily understand what the words represent. Children often enjoy listening to music; it is important to note that for music to enhance mood, research suggests the music needs to be listened to with the intention of improving mood (Ferguson & Sheldon, 2013). Caregivers can help children use music in this way by prompting them to listen to and repeat the lyrics of positive, inspiring songs when they feel upset or angry.

Similarly, it is helpful to discuss affect expression and modulation with caregivers, who are encouraged to model these skills. Caregivers can share appropriate feelings with their children (e.g. frustration at work) and encourage young children’s sharing of feelings by using reflective listening and praise when they use feeling words. With young children, reflective listening involves repeating back children’s statements verbatim, which helps them feel heard and validated. Further, caregivers are encouraged to praise children for the effective use of affect regulation strategies between sessions.

### 1.5. Cognitive coping

Cognitive coping is taught to children using non-trauma related thoughts. Children are first taught to differentiate between thoughts, feelings, and behaviours. For young children, concrete examples are most effective. Pictures of people with statements in thought bubbles can be helpful, as young children typically understand thought bubbles. To elicit thoughts, preschool children often respond better to questions such as ‘What is your brain saying?’ or ‘What are you saying to yourself?’ rather than ‘What are you thinking?’ To help children learn about behaviours (or ‘actions’), pictures of people engaged in various activities can be used.

Once children understand thoughts, feelings, and behaviours, they can be taught about the interrelationships between them. For school-age children and older, this is often explained through the depiction of the cognitive triangle. For younger children, the interrelationships of all three concepts may be too complex and therefore the relationship between thoughts and feelings and between thoughts and behaviours can be explained through coping arrows that demonstrate how our thoughts (what our brain says) influence how we feel and how our thoughts influence how we behave (act). If preschool children have difficulty identifying thoughts about a situation, thoughts can be elicited in the moment. That is, the child can be engaged in an activity (e.g. drawing shapes) and thoughts about that activity can be elicited as it happens. Then feelings and behaviours related to that activity can be discussed.

Although challenging unhelpful thoughts can be complex, young children can learn to identify unhelpful thoughts and may be able to generate more helpful replacement thoughts. One technique for practicing this with young children is reading an age-appropriate book that includes many thoughts (e.g. *Ready for Anything*; Kasza, 2009) and asking the child to identify any unhelpful thoughts in the story. The child can then try to generate more helpful thoughts for the character. This can be turned into a game in which the child, for example, uses a buzzer anytime an unhelpful thought is heard or gets a point for identifying a helpful thought. Further, young children can learn to make more positive statements to themselves, which can help them feel better. Some song lyrics and books for young children can help them develop positive self-talk that counters fearful or discouraging self-statements. *The Little Engine that Could* (Piper, 2012) and the *Can Do Duck* (Ducktor Morty, 2005) are examples of books that encourage ‘can do’ thinking.

### 1.6. Trauma narration and processing

With young children, the therapist often serves as a scribe or typist as the child dictates the trauma narrative. Prior to initiating the narrative, the therapist typically reads a developmentally-appropriate book about the trauma(s) experienced (e.g. for sexual abuse, *Please Tell*; Jessie, 1991), which helps set the stage for the child creating a book. An introductory chapter is typically included in which children provide information such as their name, age, activities they enjoy, and reason for attending therapy (e.g. ‘the not okay touch from my uncle’). Young children can be guided through this process with questions to elicit information. The completion of this chapter also provides the therapist with helpful information to set developmentally-appropriate expectations for the rest of the narrative. That is, if the child’s introductory chapter contains 3–4-word sentences (typical of preschool children), one would not expect the trauma chapters to have 6–7-word sentences. Similarly, if the introductory chapter is fairly short (also typical of young children), each trauma-related chapter is likely to be fairly short. This chapter also aids the therapist in assessing the child’s level of avoidance. If the child’s introductory chapter has multiple 3–4-word sentences and the first chapter about the trauma has two 2-word sentences, there is likely some avoidance at play.

Therapists may provide structure for the chapters using information provided early in treatment. Young children may then select the chapter to work on from two options offered by the therapist. Choices help children feel a sense of control and increase cooperation. Offering more than two options, however, can be overwhelming, especially for young children.

By age three years, children can distinguish fantasy from reality and, thus, children can be encouraged to focus on what really happened when engaged in trauma narration if they stray into fantasy. Young children may also need more prompting such as ‘What happened next?’ and prompts to include thoughts and feelings than older youth. Young children are better able to accurately respond to questions such as ‘Did that happen one time or more than one time?’ to determine the frequency of the trauma(s) rather than asking how many times the trauma(s) occurred, as a specific number offered by a young child is likely to be arbitrary (Saywitz, Lyon, & Goodman, 2011).

Although young children’s narratives are typically shorter in number of words and overall length compared to older children’s narratives, they often are able to engage in the trauma narration process and describe events that occurred with associated feelings and at least some thoughts. It is important to set developmentally-appropriate expectations that also do not underestimate a young child’s capability.

Young children’s avoidance to trauma narration can be addressed in several ways. As noted above, choices for which chapter to do next can be helpful, but even choices about seemingly small things, such as ‘Would you like to print this chapter on green paper or yellow paper?’ can help engage a child. Incorporating children’s interests as much as possible is another effective way to overcome avoidance. For example, if a child enjoys drawing, the child can illustrate the book. Although the trauma narrative is often done as a book, other mediums can be utilized, including songs, poems, talk shows, play materials, and drawings. However, children should be reminded when using dolls or puppets that their job is to demonstrate and when possible narrate what actually happened. Finding the right medium and/or planning an appealing end-of-session ritual can help children through avoidance, as they provide children with something to look forward to in the context of facing traumatic memories.

It is very important to avoid challenging children’s thoughts as they develop their narrative. Though it can be difficult to hear a child say, ‘I thought, “I am a bad boy,”’ it is best to wait until they have shared all of their inner-most thoughts as they complete the narrative before processing dysfunctional thoughts. This is important because young children are sensitive to adult responses and may censor their thoughts to fit what they think the therapist wants to hear. It is important for therapists to note overgeneralized thoughts and feelings, which are common. Young children have a natural tendency to seek out the most parsimonious explanation for any problem. For example, it is typical for young children to assume they were beaten because they are bad (as their parent said) or that mom died because of something they said such as ‘I wish you were dead.’ These thoughts are critical to address through various means including Socratic questions and behavioural experiments or evidence presented. Older children are typically able to generate alternative explanations for why trauma(s) may have occurred. However, young children often need to be presented with alternative explanations to consider. They may be engaged in conversations about, for example, reasons the adults think violence happened in their home (e.g. drug-related violence). Below is an example of a young child’s description of domestic violence as well as the Socratic questions and processing steps the therapist used to address the child’s fears of making mistakes after the narrative was completed.

#### 1.6.1. Example of processing with young children

‘Mom made a mistake. Daddy hit mommy. I cried. I was scared. I make mistakes too.’

Socratic questions …

1. Is it okay for moms to make mistakes?
2. What should happen if mom makes a mistake?
3. Do most people make mistakes?
4. Is it okay for you to make mistakes?

Young children’s thought patterns are just developing and thus they can be quite responsive to the corrective information TF-CBT provides. This is important as unchallenged maladaptive messages from perpetrators can detrimentally affect children for years. Further, encouraging optimistic and flexible thinking can lead to life-long advantages in coping with adversity and supporting future success.

Young children are adept at summarizing what they have learned in therapy for a final chapter. The final chapter may be written after they have processed the main chapters and/or after they have shared their narrative and participated in the in vivo and/or enhancing safety components. This allows them to incorporate what they have learned from those experiences in the final chapter. It should be noted, however, that the younger the child is, the more structure and guidance will be needed throughout treatment, including completing the final chapter. Below are some questions that can help young children create a final chapter.

1. Why did you come to therapy?
2. What did you learn about you? About others? About the world?
3. How did your caregiver help you?
4. What helped you feel better?
5. What would you tell other kids?
6. What are you going to be when you grow up?

### 1.7. In vivo mastery

In vivo mastery involves decreasing children’s avoidance of innocuous trauma reminders. For example, if a child experienced sexual abuse at a friend’s house and then avoids going to all friends’ homes, an in vivo plan can be created to decrease this unnecessary avoidance. Many children, however, do not develop these overgeneralized fears or overcome them during trauma narration and processing. Therefore, this component may not be needed. Fears of innocuous situations that are particularly relevant for young children are avoidance of preschool/school and of sleeping alone. For these situations, an in vivo plan can be carefully created in collaboration with the caregiver to reduce the child’s fear and resulting avoidant behaviour by gradually exposing the child to the feared (but safe) situation. The caregiver is key to the plan, as these exposures typically take place outside of session and the caregiver must commit to actively praising small steps toward the desired behaviours, while minimizing attention to avoidant behaviours. More detailed descriptions of in vivo plans are described elsewhere (e.g. Deblinger et al., 2015).

### 1.8. Conjoint sessions

Conjoint sessions are particularly helpful in reinforcing skill building and enhancing communication between child and caregiver during the initial phase of treatment. Children can teach their caregivers skills they are learning, such as focused breathing, and caregivers can practice parenting skills, such as praise and reflective listening, during brief conjoint sessions. In preparation for the sharing of the trauma narrative (if clinically appropriate), trauma-related activities can be utilized individually with the child and caregiver and later in conjoint sessions.

Toward the end of treatment, the trauma narrative can be shared in a conjoint session if deemed clinically appropriate. The caregiver should be prepared by reading the narrative and processing it with the therapist in an individual session before the conjoint session. Young children’s narratives in the form of a book are typically read by the therapist out loud; even if the young child has some emerging reading ability, the focus on reading the words rather than the content can interfere with the sharing process if reading is a struggle. For a young child who is excited to read to the caregiver, a portion, such as the introductory and final chapters, can be designated as parts the child will read, while the therapist and caregiver take turns reading other chapters.

### 1.9. Enhancing safety and future development

During this treatment component, children are taught various skills related to assertiveness and personal safety. It is important to start this component by praising children for their previous responses to the trauma, as the goal is to build on any safety skills the children already know and not make them feel as if they did an inadequate job of responding to the trauma(s) previously. The focus in this component varies depending on the trauma(s) experienced.

Among the skills taught is utilizing assertive body language. A fun exercise is for the therapist to walk across the room in a very unassertive manner (e.g. head down, slumped shoulders) and the child suggests corrections to make the therapist look more confident. Children can then practice walking assertively. During this component, it is also important for young children, especially those exposed to bullying and/or other violence, to practice expressing their needs appropriately and being assertive verbally in role plays.

Children who have experienced sexual abuse are often taught the doctors’ names for private parts (with caregiver permission). Although this is typically taught earlier in treatment, it is helpful to encourage the use of this language again as treatment nears its conclusion. Okay and not okay touches may also be reviewed at the end of treatment. As with all skills taught to children (especially young children), it is helpful to find creative and engaging ways to teach the skills. Books, videos, and other aids can be utilized to help children engage and learn the skills. One creative medium for teaching young children body ownership is the *My Body* song (www.peteralsop.com). This song can be taught to children and caregivers and sung together in session.

Young children may not understand threats so the concept of tricks can be taught to help children perceive when a situation may be not okay or dangerous. It is also helpful to teach young children the difference between a secret (something children are told they cannot ever tell anyone) and a surprise (something children do not tell for a certain amount of time and then is fun to share).

Teaching children safety skills through role plays is helpful so they practice responding to various situations. A simple phrase to help young children remember body safety skills is ‘No, Go, Tell.’ The child is taught to say ‘no,’ to try to ‘go’ and get away from an uncomfortable, confusing, or violent situation, and to ‘tell’ an adult. Each step is practiced, including a loud, assertive ‘NO!,’ moving across the room, and telling someone (the therapist or, in a conjoint session, the caregiver). Children can generate a list of trusted adults (e.g. caregiver, relatives, police, teachers) who they can tell. It is also important to emphasize that if the first adult the child tells does not help, the child should keep telling until an adult helps. This can be practiced in session by, for example, the child telling the therapist who does not respond and then the child telling the caregiver who does respond. Research has shown that practicing safety skills in role plays improves children’s learning of those skills and that the involvement of caregivers enhances children’s ability to retain and use these skills (e.g. Deblinger, Stauffer, & Steer, 2001; Finkelhor, Asdigian, & Dziuba-Leatherman, 1995).

## 2. Brief review of empirical support for TF-CBT

Research has documented the efficacy of TF-CBT in over 50 scientific studies, including 20 randomized controlled trials, the gold standard for treatment outcome research. This research has included children with various trauma experiences, including sexual abuse, physical abuse, traumatic grief, domestic and community violence, and natural disasters, as well as children in foster care and residential treatment settings (e.g. Cohen, Deblinger, Mannarino, & Steer, 2004; Dorsey et al., 2014; O’Callaghan, McMullen, Shannon, Rafferty, & Black, 2013). Overall, the results of these randomized trials document significantly greater reductions in posttraumatic stress and depressive symptoms, behavioural difficulties, and parental distress among participants in the TF-CBT condition than participants in the comparison conditions. One to two-year follow-up studies document that symptom improvements are maintained (e.g. Deblinger, Mannarino, Cohen, & Steer, 2006; Deblinger, Steer, & Lippmann, 1999). A recent study also documented significant improvements in resiliency among children treated with TF-CBT (Deblinger, Pollio, Runyon, & Steer, 2017). For a more complete review of the TF-CBT research to date, please see recently published TF-CBT treatment manuals (Cohen et al., 2017; Deblinger et al., 2015).

Early research applying TF-CBT to young children includes a study by Cohen and Mannarino (1998), which found that TF-CBT, as compared to nondirective supportive therapy, was significantly more likely to decrease PTSD, internalizing symptoms, and sexual behaviour problems among children 3–6 years with gains sustained at one-year follow up. Another study applied TF-CBT in group format with children as young as 2½ years of age (Deblinger et al., 2001). Children in the structured TF-CBT group were significantly more likely to gain knowledge and safety skills as compared to children participating in the support group condition. Moreover, the nonoffending caregivers, who were encouraged to process their children’s trauma(s) in the TF-CBT group, demonstrated significantly greater reductions in intrusive thoughts and abuse-related distress as compared to the nonoffending caregivers in the comparison condition.

More recent studies applying TF-CBT to young children also documented the model’s efficacy with this population. These investigations have involved a range of traumas, including sexual abuse (Deblinger, Mannarino, Cohen, Runyon, & Steer, 2011), domestic and community violence, natural and man-made disasters, neglect, and traumatic grief (CATS consortium, 2010; Kameoka et al., 2015; Lyons, Weiner, & Scheider, 2006; Murray et al., 2015; Salloum et al., 2016).

## 3. Conclusions/summary

Young children and their caregivers are highly responsive to TF-CBT. To implement TF-CBT with this population most effectively, it is important to consider children’s developmental level and implement the model accordingly. The clinical strategies described above actively engage nonoffending caregivers and incorporate picture books, play, drawing, singing, role playing, and other creative activities in structured ways to implement the PRACTICE components of TF-CBT with young children and enhance their responsiveness to treatment in the aftermath of trauma.
